# Pilin Processing Follows a Different Temporal Route than That of Archaellins in *Methanococcus maripaludis*

**DOI:** 10.3390/life5010085

**Published:** 2015-01-05

**Authors:** Divya B. Nair, Ken F. Jarrell

**Affiliations:** Department of Biomedical and Molecular Sciences, Queen’s University, Kingston, ON K7L 3N6, Canada; E-Mail: 7ndb@queensu.ca

**Keywords:** archaellins, pilins, signal peptide, N-linked glycosylation, archaea

## Abstract

*Methanococcus maripaludis* has two different surface appendages: type IV-like pili and archaella. Both structures are believed to be assembled using a bacterial type IV pilus mechanism. Each structure is composed of multiple subunits, either pilins or archaellins. Both pilins and archaellins are made initially as preproteins with type IV pilin-like signal peptides, which must be removed by a prepilin peptidase-like enzyme. This enzyme is FlaK for archaellins and EppA for pilins. In addition, both pilins and archaellins are modified with N-linked glycans. The archaellins possess an N-linked tetrasaccharide while the pilins have a pentasaccharide which consists of the archaellin tetrasaccharide but with an additional sugar, an unidentified hexose, attached to the linking sugar. In this report, we show that archaellins can be processed by FlaK in the absence of N-glycosylation and N-glycosylation can occur on archaellins that still retain their signal peptides. In contrast, pilins are not glycosylated unless they have been acted on by EppA to have the signal peptide removed. However, EppA can still remove signal peptides from non-glycosylated pilins. These findings indicate that there is a difference in the order of the posttranslational modifications of pilins and archaellins even though both are type IV pilin-like proteins.

## 1. Introduction

The mesophilic methanogen *Methanococcus maripaludis* is one of the best studied Archaea with regards to both N-linked glycosylation and surface structures [1,2,3,4]. Part of the reason for this is the development of a number of genetic tools that have made this methanogen a model organism for archaeal biology [5,6,7,8,9].

*M. maripaludis* possesses two surface structures that have been studied, namely the archaella (formerly archaeal flagella [10] and type IV pili [2,11,12,13,14]). Both of these structures are believed to be assembled via a bacterial type IV pilus mechanism [2,15,16]. Indeed, the many similarities of archaella to bacterial type IV pili and their lack of homology to bacterial flagella, other than their shared involvement in swimming motility, initially led to the proposal of a distinct name for the archaeal structure [10]. Both archaella and archaeal type IV pili are assembled from structural proteins synthesized initially as preproteins with a class III (type IV pilin-like) signal peptide which is subsequently cleaved by a dedicated prepilin peptidase-like enzyme (signal peptidase III [17,18,19,20,21,22,23,24]). Also shared with type IV pili systems of bacteria is the presence of an essential ATPase involved in polymerization of the subunits into a filament and a conserved membrane platform protein thought to interact with the ATPase [25,26,27,28,29,30,31,32].

In *M. maripaludis*, the archaella are composed of three structural glycoproteins (the archaellins, FlaB1, FlaB2 and FlaB3) that are all modified at multiple positions with an N-linked tetrasaccharide [30,33,34]. The prepilin peptidase-like enzyme responsible for signal peptide removal from archaellins was identified as FlaK and its activity shown to be necessary for archaella assembly [19,35]. Gene deletion studies, coupled with mass spectrometry of purified archaella filaments, have identified a number of enzymes in both the assembly of the glycan (glycosyltransferases, oligosaccharyltransferase [34]) and in the biosynthetic pathways for the individual sugar components [36,37,38]. Electron microscopy studies have further demonstrated that interference with the N-linked glycosylation pathway has severe effects on archaellation. In mutants of *M. maripaludis* where the glycan structure has been truncated from the tetrasaccharide to less than two sugars, cells cannot assemble archaella [34].

Less numerous and thinner than archaella are the Epd pili of *M. maripaludis* [13]. At least five different type IV pilin-like genes (*epdA, epdB, epdC, epdD* and *epdE*) have been shown by deletion analysis coupled with electron microscopy to be necessary for normal piliation [12,14], with *epdE* (*mmp1685*) shown by mass spectrometry analysis of purified pili to encode the major structural subunit [12]. Mass spectrometry analysis of purified pili also revealed that EpdE was a glycoprotein with multiple sites occupied by an N-linked glycan [12]. Unexpectedly, however, the attached glycan was not identical to the tetrasaccharide of the archaellins [33] but instead was a pentasaccharide that had an unidentified hexose attached as a branch to the linking sugar of the archaellin tetrasaccharide [12]. In addition, EpdE had an unusual electrophoretic mobility in SDS-PAGE. The mass of EpdE predicted from its mature protein sequence was 6400 Da while an intact mass calculated from mass spectrometry was 9700 Da, suggesting modification of the protein with glycan at three sites. However, SDS-PAGE determination of the mass of EpdE from purified pili was 15,000−17,000 Da [12].

Unusually, in *M. maripaludis*, the pilins have their own dedicated prepilin peptidase, EppA, which cleaves the signal peptide from pilins but not archaellins [21]. This is in contrast to other studied Archaea which have a single prepilin peptidase-like enzyme, PibD, that cleaves all type IV pilin-like proteins, including archaellins, pilins and sugar binding proteins [20,22,23,24]. For *M. maripaludis*, it was shown by swapping experiments of the −2 to +2 amino acids around the conserved cleavage site that mutant archaellins that contained the −2 to +2 amino acids found in pilins could be processed by EppA [21]. Similar switching of the amino acids found at these sites in archaellins to pilins did not result in pilins that could be processed by FlaK. However, previous work in *Methanococcus voltae*, a close relative of *M. maripaludis*, established that the +3 glycine of the archaellin mature protein was essential for signal peptide removal in that methanogen [39]. The failure of the −2 to +2 archaellin to pilin amino acid swap to lead to a hybrid pilin that could be processed by FlaK may be thus explained, since *M. maripaludis* pilins do not possess glycine at the +3 position [12,14,21]. Here, we investigated whether a simple change of the +3 amino acid of the pilin EpdE to a glycine might make it susceptible to processing by FlaK. During the course of these studies, it became evident that pilins were not glycosylated unless they were first processed by EppA. This is in contrast to archaellins where it was shown that signal peptide-bearing archaellins of a *flaK* deletion strain were still glycosylated. Hence, it seems that pilins follow a different order of posttranslational modification that is unlike that of the other type IV pilin-like proteins in *M. maripaludis*, namely the archaellins.

## 2. Materials and Methods

### 2.1. Strains and Growth Conditions

*Methanococcus maripaludis* MM900 [7] and various single and double deletion mutants derived from it were used in this study (Table 1). These mutants include; *∆flaK*, a non-archaellated strain deleted for the pre-archaellin peptidase [35], *∆eppA*, a non-piliated strain deleted for the prepilin peptidase [11]; *∆aglB*, a strain deleted for the oligosaccharyltransferase necessary for N-glycosylation [34] and the double mutants, *∆flaK∆eppA* [11], *∆flaK∆aglB* and *∆eppA∆aglB*, the latter two created in this study. All strains were routinely grown in Balch medium III [40] at 35 °C under a headspace gas of CO_2_/H_2_ (20/80). For steps of the inframe mutant generation procedure as described below, cells were grown in McCas medium [7]. In complementation experiments, transformants were selected by the addition of puromycin (2.5 µg/mL) to Balch medium III. *Escherichia coli* TOP10 cells (Invitrogen), used for various cloning steps, were grown in Luria-Bertani medium supplemented with ampicillin (100 µg/mL) as needed. *E. coli* strain BL21 (DE3)/pLysS, grown in Luria-Bertani medium supplemented with ampicillin (100 µg/mL) and chloramphenicol (30 µg/mL), was used as host for the overexpression of a C-terminal histagged version of EpdE.

**Table 1:** Strains and plasmids used in this study.

	Description and/or genotype	Source or reference
**Strains**		
*E. coli* K113	BL21(DE3)/pLysS; expression host, Cm^R^	Novagen
*Methanococcus maripaludis* Mm900	Δ*hpt*	[7]
*M. maripaludis ∆flaK*	Mm900*∆flaK*	[35]
*M. maripaludis ∆eppA*	Mm900*∆eppA*	[11]
*M. maripaludis ∆flaK ∆eppA*	Mm900*∆flaK ∆eppA*	[11]
*M. maripaludis ∆aglB*	Mm900*∆aglB*	[34]
*M. maripaludis ∆flaK ∆aglB*	Mm900*∆flaK ∆aglB*	This study
*M. maripaludis ∆eppA ∆aglB*	Mm900*∆eppA ∆aglB*	This study
**Plasmids**		
pKJ574	pCRPrtNeo with inframe deletion of *aglB*	[34]
pKJ697	pCRPrtNeo with inframe deletion of *eppA*	[11]
pET23a+	T7 promoter-based expression vector, Amp^r^	Novagen
pKJ900	pET23a+ with *epdE* *Nde1-Xho1* fragment C-terminal histagged	This study
pWLG40	hmv promoter-lacZ fusion plus Pur^r^ cassette; Amp^r^	[30]
pKJ880	pWLG40 with *epdE* complement	[12]
pKJ1072	pWLG40 with *epdE* C-terminal histagged complement	This study
pKJ1079	pWLG40 with *epdE* (+3 Gly) C-terminal histagged complement	This study
pKJ1107	pWLG40 with *epdE* C-terminal FLAG complement	This study
pKJ1108	pWLG40 with *epdE* (+3 Gly) C-terminal FLAG complement	This study
pHW40	*nif* promoter-lacZ fusion plus Pur^r^ cassette; Amp^r^	[30]
pKJ1169	pHW40 with *epdD* C-terminal FLAG complement	This study
pKJ1226	pHW40 with *epdE* C-terminal FLAG complement	This study
pKJ1216	pHW40 with +1 Ala +3 Gly *epdE* C-terminal FLAG complement	This study
pKJ711	pHW40 with *epdE*	[13]

### 2.2. M. maripaludis Mutant Generation

Plasmid pKJ574 [34] was used for the generation of an inframe deletion of *aglB* in the ∆*flaK* strain [35] and plasmid pKJ697 [11] for the generation of an *eppA* deletion in the pre-existing ∆*aglB* strain [34] to create markerless double mutants, using procedures as previously described [7,36]. Plasmids were transformed into *M. maripaludis* strains using the PEG precipitation method [6]. Individual transformant colonies that grew on McCas plates containing hypoxanthine were picked and inoculated into Balch medium III. Deletion mutants were identified by using washed whole cells resuspended in 2% (w/v) NaCl as template for PCR along with sequencing primers (Table 2) designed to amplify across the targeted gene deletion. The PCR products were examined by agarose gel electrophoresis and the size compared to that predicted for the wildtype and the deletion version of the targeted gene. Transformants that generated the predicted deletion size PCR product were restreaked onto Balch medium III plates and single colonies picked and again screened by PCR to confirm their purity. The pre-existing deletions of *flaK* and *aglB* in these double mutants were also confirmed by PCR using the sequencing primers listed in Table 2.

**Table 2:** Primers used in this study *.

Primers	Primer Sequence 5’ to 3’	Restriction site
1685+3_sdm_For Nsi1	CCAATGCATGAAATTTTTAGAAAAACTAACATCAAAAAAAGGTC AAATAG*G*AATGGAACTCGG *	Nsi1
1685+1+3_sdm_For Nsi1	CCAATGCATGAAATTTTTAGAAAAACTAACATCAAAAAAAGGT*G* *C*AATAG*G*AATGGAACTCGG *	Nsi1
1685_Mlu_Rev	AGCACGCGTTTAATCCGTAATATTTGACATTTGTGAGG	Mlu1
1685_Nsi1_For	CCAATGCATGAAATTTTTAGAAAAACTAACATC	Nsi1
1685_histag_Mlu_Rev	CGCGACGCGTTTAGTGATGGTGGTGATGGTGATCCGTAATA TTTGACATTTGTGAGG	Mlu1
1685_FLAG_Mlu_Rev	CGACGCGTTTATTTGTCATCGTCATCTTTGTAATCATCCGTA ATATTTGACATTTGTGAG	Mlu1
1685_For_exp	GGAATTCCATATGATGAAATTTTTAGAAAAACTAACATC	NdeI
1685-Rev_exp	CCGCTCGAGATCCGTAATATTTGACATTTGTGAG	XhoI
1283_Nsi1_For	CCAATGCATGTCTGTTGCTTTAAAGAAGTTTTTTTCGAAACG	Nsi1
1283_FLAG_Mlu_Rev	GCTACGCGTTTATTTGTCATCGTCATCTTTGTAATCACTAACTT CACTTAAAGCATCTAT	Mlu1
FlaK_seq_For	AATATCTGGCGGATACAGG	-
FlaK_seq_Rev	TTCAAAGCCAATAGATACTGC	-
EppA_seq_For	CTGGAGCTGTATGAAATGCAAC	-
EppA_seq_Rev	GGATGACCTGGGATAATGCAGG	-
AglB_seq_For	CATAACCATATTTGTAATTAAC	-
AglB_seq_Rev	CTCAATAGCCATAAAATCACC	-

* Restriction enzyme sites added to primers are underlined. SDM changes are shown in bold italics.

### 2.3. Complementation Experiments

Plasmid pKJ711, containing *eppA* in pHW40 under the control of the *nif* promoter [41], was used to complement the ∆*eppA* mutant, as reported previously [13].

### 2.4. Expression of Epitope-Tagged Versions of EpdD and EpdE in Various M. maripaludis Strains

For all PCR reactions, washed whole cells of *M. maripaludis* MM900 were used as template. PCR using primers 1685+3_sdm_For_Nsi1 and either 1685_histag_mlu_rev or 1685_FLAG_Mlu_Rev was used to change the +3 position (relative to the prepilin peptidase cleavage site) of EpdE from alanine to glycine. The long forward primer (Table 1) was designed to include the desired change while the reverse primer was used to create either a Histag or FLAG tag at the C-terminus. NsiI and MluI restriction sites were incorporated into the forward and reverse primers, respectively, to facilitate cloning steps. The PCR product was digested with NsiI and MluI and cloned into pWLG40, where the expression of the cloned gene is driven by the strong constitutive *hmv* promoter [42]. This created pKJ1079 for the His-tagged version of EpdE with the +3 glycine change and pKJ1108 for the FLAG-tagged version (Table 1). Successful complementation of a ∆*epdE* strain with a wildtype copy of the *epdE* gene (*mmp1685*) cloned into this vector was previously demonstrated [12]. Similarly, using primer 1685+1+3_sdm_ForNsi1 and reverse primer 1685_FLAG_Mlu_Rev, a mutant version of EpdE which had its +1 amino acid changed to alanine and its +3 amino acid changed to glycine was generated and cloned into pHW40 creating pKJ1216. In the pHW40 vector, the transcription of the cloned gene is under the control of the inducible *nif* promoter [41].

A C-terminal histagged version of wildtype EpdE was generated by PCR using primers 1685_NsiI_For and 1685_histag_Mlu_Rev. The PCR product was digested with NsiI and MluI and cloned into the vector pWLG40 creating pKJ1072. Similarly, a C-terminal FLAG tagged version of EpdE was amplified by PCR using primers1685_NsiI_For and 1685_FLAG_Mlu_Rev and cloned into pWLG40, creating pKJ1107 or into pHW40, creating pKJ1226. A C-terminal FLAG tagged version of the minor pilin EpdD [14] was generated by PCR using primers 1283_NsiI_For and 1283_FLAG_Mlu_Rev and the product cloned into pHW40, creating pKJ1169. The various plasmids carrying epitope-tagged versions of the pilin genes (Table 1) were transferred into the wildtype and mutant strains via the PEG procedure [6].

### 2.5. Overexpression and Purification of His-Tagged EpdE

The *epdE* gene was amplified by PCR from wildtype cells using primers 1685-For-exp and 1685-Rev-exp (Table 2). The PCR product was purified, digested with NdeI and XhoI and cloned into the expression vector pET23a+ generating a C-terminal histagged fusion. This plasmid, designated pKJ900, was subsequently electroporated into the expression strain *E. coli* BL21 (DE3)/pLysS. Purification of EpdE from a crude membrane fraction of cells induced with IPTG for 2 h was carried out using nickel affinity purification under denaturing conditions by using a His-bind kit (Novagen), as directed by the manufacturer. Purified protein was concentrated and used to raise antibodies in chickens [30] (RCH Antibodies, Sydenham, Ontario Canada). The anti-EpdE antibodies were further subjected to affinity purification. Purified Histagged EpdE was electrophoresed through a 1.5 mm thick gel (15%) and blotted [43] to supported nitrocellulose (0.22 μm, GE Water and Process Technologies). Transferred protein was located on the membrane by Ponceau S staining [44]. This region was cut into small pieces and blocked overnight at 4 °C, washed and then incubated with 1 mL crude antibody at 4 °C overnight. After extensive washing, the antibodies were released from the membrane by a 5 min incubation in 500 µL 0.2 M glycine, pH 2.8, followed by immediate neutralization using 125 µL 1 M Tris-HCl, pH 8. These antibodies were used at 1:100 dilution in Western blots.

### 2.6. Western Blot Analysis 

*M. maripaludis* whole-cell lysates were prepared by centrifuging 1 mL of an overnight culture for 1 min in a mini-centrifuge. The pellets were resuspended in 50–100 µL of 2% NaCl and an equal volume of 2× electrophoresis sample buffer was then added and the samples boiled for 5 min. Samples were electrophoresed through 15% (FlaB2 blots) or 17.5% (pilin blots) SDS-PAGE and then transferred to an Immunobilon-P membrane (Millipore, Bedford, MA, USA) [43]. Prestained protein ladders were used as molecular mass standards (Page Ruler Prestained Protein Ladder or Page Ruler Plus Prestained Protein Ladder; ThermoFisher Scientific, Waltham, MA, USA). Major archaellin FlaB2 was detected with chicken anti-FlaB2 specific antibodies [38]. Major pilin EpdE was detected with chicken anti-EpdE specific antibodies, affinity purified as described above. Horseradish peroxidase-conjugated rabbit anti-chicken immunoglobulin Y (Jackson Immuno Research Laboratories, West Grove, PA, USA) was used as secondary antibody. His-tagged proteins were detected using a rabbit polyclonal IgG against the hexahistidine tag (Santa Cruz, Biotechnology, Dallas, TX, USA) as the primary antibody and horseradish peroxidase-conjugated goat anti-rabbit IgG (BioRad Laboratories, Mississauga, ON, Canada) as the secondary antibody. To detect FLAG-tagged proteins, a rabbit polyclonal anti-FLAG primary antibody (SIGMA-Aldrich, St. Louis, MO, USA) and a horseradish peroxidase-conjugated goat anti-rabbit IgG secondary antibody (BioRad Laboratories) were used. All blots were developed with a chemiluminescent kit according to the manufacturer’s instructions (Roche Molecular Biochemicals, Laval, QC, Canada).

## 3. Results and Discussion

### 3.1. The +3 Position Alone Does Not Distinguish Archaellins from Pilins as FlaK Substrates

**Figure 1:**
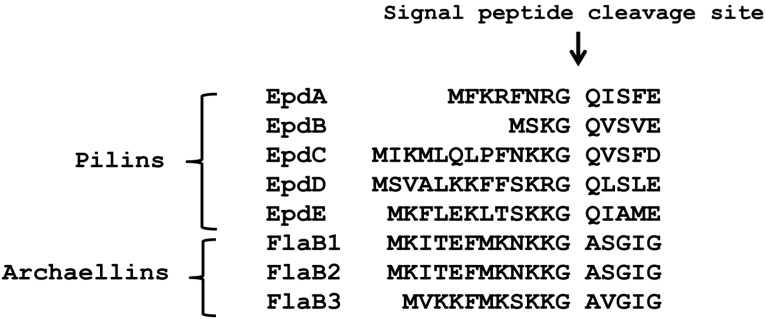
Alignment of the N-terminus region, including the signal peptide, of type IV pilin-like proteins of *M. maripaludis*.

*M. maripaludis* has two prepilin peptidase-like enzymes. In addition to the one that processes archaellins, identified previously by Bardy and Jarrell [18] and designated FlaK, Szabo *et al.* [21] discovered EppA which was shown to process the pilins EpdA and EpdC. Both pilins and archaellins share significant amino acid sequence similarities in their signal peptides, including key conserved residues near the cleavage site itself (positions −1 to −3; Figure 1), and in the mature N-terminus [2,3,39].

**Figure 2:**
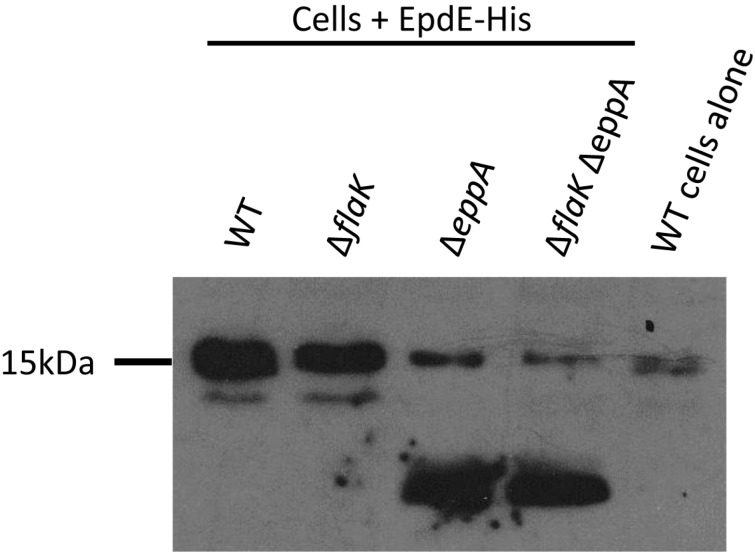
Western blot analysis of various *M. maripaludis* mutant strains expressing a plasmid borne C-terminal histagged version of EpdE. Whole cell lysates were separated by SDS-PAGE (17.5% gel), transferred to Immobilon membrane and developed with antibodies to the Histag. Note the 16 kDa band that is present in wildtype cells without the vector.

Nonetheless, FlaK can only process archaellins and EppA only pilins [21]. Changing only the four amino acids, the −2 to +2 positions around the signal peptide cleavage site, from those normally found in an archaellin to those of a pilin, was enough to transform the mutant archaellin into a substrate for EppA [21]. A similar change of the −2 to +2 amino acids normally found in a pilin to those found in an archaellin did not allow FlaK to cleave the modified pilin. However, it had been previously shown that in *M. voltae* that changing the +3 glycine to valine in an archaellin prevented cleavage of the signal peptide in *in vitro* experiments [39]. Since glycine is universally found in the +3 position in archaellins [45], we reasoned that the failure of the initial swapping experiment might be because the hybrid pilin carrying the −2 to +2 archaellin amino acids still retained the pilin +3 position, which is not glycine. Thus, we used a PCR-based protocol to change the +3 position of EpdE from alanine to glycine to investigate whether this single amino change would then make the mutant EpdE pilin a substrate for FlaK. Our intention was to express both the wildtype version and the +3 glycine version of EpdE as C-terminal histagged versions from a plasmid in wildtype cells and in mutants deleted for *eppA*, *flaK* or both *eppA* and *flaK* and develop Western blots with anti-His antibodies. However, complications arose using the histagged pilin, as shown in Figure 2 for strains expressing the wildtype version of EpdE-His. In the two mutant strains carrying a deletion of *eppA* (∆*eppA* and ∆*flaK*∆*eppA*), the majority of the crossreacting material in Western blots appeared as a faster migrating band that was not seen in strains that still possessed *eppA* (Figure 2). In all four strains with the vector, a band was observed at approximately 16 kDa, the size expected for EpdE [12]. This band was much weaker in the strains deleted for *eppA*. However, a band of similar mobility and intensity was detected even in control cells that had not received the plasmid. This is likely to be the 16.1 kDa sirohydrochlorin cobaltochelatase which has 12 histidine residues in a 15 amino acid stretch [30].

As this naturally occurring cross-reacting band complicated interpretation of the pilin processing, the EpdE constructs were remade with a C-terminal FLAG-tag and transformed into the wildtype and mutant strains. Western blots developed to detect this epitope tag indicated that the electrophoretic mobility of the FLAG-tagged wildtype EpdE was the same in both wildtype cells and the *flaK* mutant and at a position expected from the molecular mass (about 16 kDa) of EpdE found in isolated pili samples [12], with a second band also detected at a lower apparent molecular mass (about 12 kDa). However, in mutants where *eppA* was deleted (∆*eppA* and ∆*eppA*∆*flaK* strains), no EpdE was detected at the 16 kDa position in Western blots but instead only a faster migrating band was observed (Figure S1). This resulted in an extremely large reaction with the anti-FLAG antibodies at about 12 kDa even though corresponding Coomassie stained gels did not reveal any obvious differences in the intensity of protein bands at this molecular mass in the four lanes (not shown). However, it has been previously reported that pilins from isolated pili (mainly EpdE) do not stain well with Coomassie Blue [12]. Despite the large signal at 12 kDa, no crossreacting band was observed at 16 kDa in strains deleted for *eppA*. This Western blot result may be due to excess EpdE being synthesized from the *hmv* promoter accumulating in the membrane. The reduction in apparent molecular mass of EpdE in the mutants lacking EppA was a striking and unexpected observation since, in the strains deleted for *eppA,* the signal peptide would still be attached to EpdE and the protein would be 12 amino acids larger. The same pattern was observed when the +3 position of EpdE was changed to glycine and expressed in the various mutant backgrounds (Figure S1). The appearance of this faster migrating species is indicative of a pilin which has not had its signal peptide removed. Since EpdE is known to be a protein modified with an N-linked pentasaccharide at multiple sites [12], a logical explanation for the apparent sizes of EpdE in the various mutants is that the EpdE protein is not N-glycosylated unless the signal peptide is first removed by EppA. The non-glycosylated EpdE would migrate as a much lower molecular mass protein than the glycosylated version. This hypothesis is investigated further below.

These results indicated that the single amino change at the +3 position to glycine was not sufficient to turn the EpdE pilin into a FlaK substrate since if FlaK was now able to process this mutant version of EpdE, then in the ∆*eppA* mutant which still possesses *flaK*, EpdE would have had its signal peptide removed and then be glycosylated and migrate on the Western blot at about 16 kDa. We then created a version of EpdE-FLAG that had both the +1 and +3 amino acids changed to those found in FlaB2. For these experiments, we expressed the mutant proteins from the *nif* promoter, expecting this would lead to less protein being produced, and cleaner Western blots than those experienced with the *hmv* produced pilins, as was in fact evident. This new mutant pilin now possessed the −2, −1, +1 and +3 amino acids found in archaellins but expression of this EpdE variant in the various mutants still indicated it was not a substrate for FlaK, despite possessing the potential FlaK-processing site (Figure S2). We did not change the +2 position in these experiments as it is one of the most variable in the N-terminus of archaellins [46], and in both *M. maripaludis* and *M. voltae* there are examples of archaellins bearing serine, valine or threonine at this position. Since two of the pilins (EpdB and EpdC) of *M. maripaludis* also possess valine at +2, this position does not seem to be important in distinguishing substrate specificity of the two prepilin peptidases. The apparent mass of the mutant EpdE-FLAG was about 12 kDa in all three mutant strains as well as in the wildtype strain, indicating that this version of EpdE was not processed by either prepilin peptidase. This would suggest that the +1 glutamine is essential for EppA processing [21] since this position was now changed to alanine. The recognition sites for FlaK clearly extend further from the cleavage site than for EppA.

### 3.2. Posttranslational Modifications of Native EpdE in Various Mutant Strains 

To examine the unusual finding regarding the two posttranslational modifications of the pilins more thoroughly, we raised antibodies to EpdE and looked at the size of native EpdE in wildtype cells and the prepilin peptidase mutants. EpdE was expressed as an N-terminally His-tagged protein using a pET vector system and subsequently purified from induced *E. coli* cells using Ni-affinity chromatography (Figure S3). The estimated mass of the purified EpdE was about 12,000 Da, much greater than the mass calculated from the protein sequence itself, despite the fact that the *E. coli* version would be non-glycosylated. This is consistent with the aberrant electrophoretic mobility of the EpdE subunits incorporated into pili [12]. The purified protein was used to raise antibodies in chickens.

**Figure 3:**
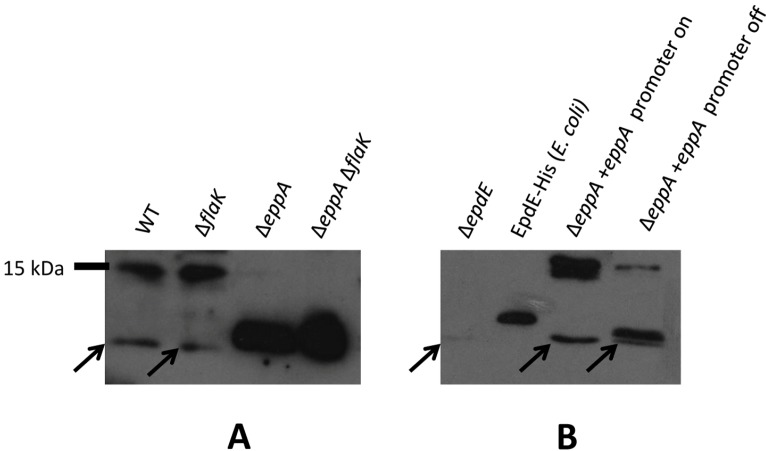
Western blot detection of native EpdE in various mutant backgrounds. (**A**) Western blot analysis to detect EpdE in various *M. maripaludis* mutant strains using anti-EpdE antibody. (**B**) Detection of EpdE in Western blots of the ∆*eppA* mutant and in the ∆*eppA* mutant following complementation with a plasmid borne copy of *eppA*. Shown are samples after the complemented cells were grown for three transfers in N-free medium supplemented with alanine (promoter on conditions) and the same cells grown in N-free medium supplemented with NH_4_Cl (promoter off-conditions). C-terminal histagged EpdE was expressed in *E.coli* and purified by a Ni-affinity column is used for size comparison. In both (A) and (B), whole cell lysates were separated by SDS-PAGE (17.5% gel), transferred to Immobilon membrane and developed with antibodies to EpdE. Arrows point to a nonspecific cross-reactive band that can be observed in all whole lysate lanes, including the ∆*epdE* lane.

Western blots using the purified IgY were not specific, however, requiring a subsequent affinity purification step. Antibodies eluted from this step were used in subsequent Western blots. The anti-EpdE antibodies, with their affinity purified from EpdE protein expressed in *E. coli* where it would not be glycosylated, may react more strongly to non-processed pilin compared to the wildtype pilin glycoprotein. As seen initially with the EpdE FLAG-tagged protein expressed in trans, native EpdE in *eppA* deletion strains also migrated as a much smaller protein than in wildtype cells or the *flaK* deletion strain (Figure 3A). When the ∆*eppA* strain was complemented with a plasmid borne copy of *eppA*, the EpdE detected in Western blots returned to the size observed in the wildtype cells (Figure 3B), proving that EpdE is, in fact, an EppA substrate, as initially predicted by Szabo *et al.* [21] and that signal peptide removal was necessary for the dramatic shift of the pilin to the higher apparent molecular mass. For a size comparison, we also included the purified C-terminal histagged version of EpdE expressed in *E. coli* and used to generate antibodies. This version of EpdE would be non-glycosylated and still have its signal peptide attached, as *E. coli* lacks the genes necessary for the two posttranslational modifications. It would however be larger than the native EpdE protein by virtue of the extra six C-terminal histidine residues. The size of EpdE as detected by Western blots using anti-EpdE antibodies is smaller in the ∆*eppA* and ∆*eppA* ∆*flaK* strains than the *E. coli*-produced histagged version. This would be consistent with the ∆*eppA* and ∆*eppA* ∆*flaK* strain versions being non-glycosylated and with their signal peptide attached, as in the *E. coli* produced version but without the hexahistidine C-terminal extension.

### 3.3. Signal Peptide Removal Is a Prerequisite for N-glycosylation of Pilins but Not Archaellins

To explore further the order of the signal peptide removal and N-glycosylation modifications of archaellins and pilins, we looked at Western blots of the two types of proteins in cells in which the signal peptide removal or N-glycosylation or both was prevented. Prevention of glycosylation was obtained by using a ∆*aglB* strain since AglB is the oligosaccharyltransferase responsible for the terminal step in the N-linked glycosylation pathway [1,34,47]. For analysis of the archaellin FlaB2, a ∆*flaK∆aglB* double deletion strain was created, using the ∆*flaK* mutant as the starting strain. PCR analysis confirmed that the mutant was deleted for both *flaK* and *aglB* (Figure S4).

**Figure 4:**
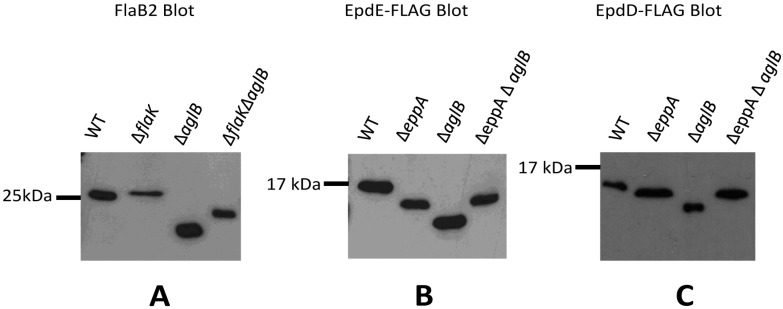
Examination of posttranslational modifications of archaellins and pilins in various mutant backgrounds. (**A**) Western blot detection of archaellin FlaB2 in various *M.* *maripaludis* mutant strains. Whole cell lysates were separated by SDS-PAGE (15% gel), transferred to Immobilon membrane and the blot developed with antibodies to FlaB2. (**B**) Western blot detection of EpdE in various *M. maripaludis* mutant strains expressing a plasmid borne C-terminal FLAG-tagged version of EpdE. (**C**) Western blot detection of EpdD in various *M. maripaludis* mutant strains expressing a plasmid borne C-terminal FLAG-tagged version of EpdD. For panels (B) and (C), whole cell lysates were separated by SDS-PAGE (17.5% gel), transferred to Immobilon membrane and developed with antibodies to the FLAG-tag.

FlaB2 was detected in Western blots of whole cell lysates of wildtype, ∆*flaK*, ∆*aglB* and the ∆*flaK∆aglB* double mutant strains (Figure 4A). FlaB2 runs as a slightly larger protein in the ∆*flaK* mutant compared to wildtype, as a result of it retaining the 12 amino acid signal peptide [18,19]. In a ∆*aglB* mutant, where N-glycosylation at four sequons is now prevented [34], the protein migrates much faster than in the wildtype cells as expected from the lack of attached glycan. In the ∆*flaK∆aglB* double mutant, the FlaB2 detected is slightly larger in molecular mass than that observed in the ∆*aglB* mutant. This band represents the non-glycosylated FlaB2 with its signal peptide intact, meaning that the smaller molecular mass band observed in the ∆*aglB* mutant would be the non-glycosylated protein but with its signal peptide removed. Thus, for archaellins, the two posttranslational modifications can occur independently of the other; N-linked glycosylation of the protein can occur whether the signal peptide is removed or not and it is also possible for the protein to have its signal peptide removed whether the protein is first glycosylated or not.

We then turned our attention to signal peptide processing and N-glycosylation of the pilins in appropriate mutant strains. For this, we examined both EpdE as well as EpdD (MMP1283), a recently identified minor pilin essential for pili formation in *M. maripaludis* [14]. In addition to the ∆*eppA* and *∆aglB* strains, a ∆*eppA ∆aglB* double mutant was constructed for this work using the *∆aglB* strain as the starting strain for a deletion of *eppA*. PCR screening of transformants identified the double mutant (Figure S4B). In these experiments, we created a FLAG-tagged version of EpdE and EpdD expressed from the regulatable *nif* promoter of pHW40 which we transformed into the various strains. For both EpdE and EpdD, the pattern obtained in Western blots was the same. In the ∆*eppA* mutant where the signal peptide would not be removed from the pilins, the protein migrated faster than the same protein in wildtype cells (Figure 4B,C), despite the fact the protein would be larger by the additional signal peptide in the ∆*eppA* strain. In the ∆*aglB* mutant, both EpdE and EpdD migrated even faster than in the ∆*eppA* strain. This indicated for the first time that the one possible N-linked glycosylation site in EpdD is, in fact, occupied in wildtype cells and that AglB is necessary for this N-glycan attachment. EpdE was already known to have an attached N-linked glycan [12]. In the ∆*aglB*∆*eppA* double mutant, the electrophoretic mobility of EpdE-FLAG and EpdD-FLAG was the same as it was in the ∆*eppA* strain. The pilin band observed in Western blots of the ∆*eppA* mutant most likely represents a non-glycosylated pilin with its signal peptide still attached since the electrophoretic mobility of the pilins in the ∆*eppA* ∆*aglB* double mutant is the same, and glycosylation would be prevented in the double mutant. In the ∆*aglB* mutant alone, the EpdE-FLAG and EpdD-FLAG migrates as a smaller protein than in the ∆*eppA* ∆*aglB* mutant, consistent with this pilin having its signal peptide removed in addition to being non-glycosylated. This indicates that EpdD is an EppA substrate, as predicted previously [21]. Thus, in the case of pilins, the addition of the N-glycan to pilins is dependent on the signal peptide first being cleaved. This seems to be an unusual requirement as this is not the case with archaellins and, in *P. aeruginosa*, O-glycosylation of type IV pilins was shown to occur in a *pilD* mutant indicating that signal peptide removal was not a prerequisite for glycosylation in this system either [48]. On the other hand, signal peptide removal can occur even if the pilins are nonglycosylated, as observed in archaellins, and as originally shown for *M. maripaludis* minor pilins EpdA and EpdC in studies where these pilins and EppA were co-expressed in *E. coli* [21]. In that study, the prepilins were non-glycosylated because *E. coli* lacks the N-glycosylation pathway and yet EppA was still able to process the proteins.

The presence in one organism of two different proteins having different N-linked glycans in Archaea is rare but occurs for the pilins and archaellins of *M. maripaludis* [12,33]. In *M. voltae*, the same trisaccharide glycan has been shown to be attached to both archaellins and the S-layer protein [49]. In *Hfx. volcanii*, the same *agl* genes involved in formation of the glycan initially reported to be attached to the S-layer glycoprotein [50] were also found to be involved in the N-glycosylation of archaellins [51], although it is unknown whether the glycan is identical. In different extreme halophiles, the same protein has been shown to be modified with two different N-linked glycans. This was first reported in the S-layer protein of *Halobacterium salinarum* where there is a single site which has an attached repeating unit N-linked glycan and 10 sites which contain a sulfated oligosaccharide [52,53]. Interestingly, archaellins in *Hb. salinarum* are modified with the sulfated oligosaccharide but not with the repeating unit glycan [54,55,56]. Recently, in *Hfx. volcanii,* two different N-linked glycans have been found on the S-layer protein when cells are cultured in medium containing reduced salt concentrations [57]. How the cell delivers the different glycan to the different sites on the same protein is unknown but critically in the *Hfx volcanii* case, the transfer of the low salt glycan has been shown to occur independently of the lone identified oligosaccharyltransferase [57]. This is not the case in *M. maripaludis* where the glycan attached to both archaellins and pilins depends upon the presence of AglB, since, in a ∆*aglB* mutant, both archaellins and pilins migrate as much smaller proteins indicative of being non-glycosylated. How the pilins get decorated with a pentasaccharide glycan while the archaellins are modified with a tetrasaccharide is currently unknown.

In some bacteria, appendages are localized in defined regions, such as the cell pole [58,59,60,61]. Type IV pili are polar organelles in *Pseudomonas aeruginosa* [60]*,* for example, and in *Caulobacter crescentus* flagella, pili, the stalk and holdfast all assemble at the so-called polar region of the cell, thought to be a distinct functional and biochemical entity [62,63]. In such situations, it is possible for the cell to differentially locate specific proteins to the cell pole [60,61,62,63,64,65]. For *M. maripaludis*, it could then be possible for a localized pilus-specific assembly apparatus to exist that might contain, among other proteins, EppA and a unique N-glycosylation pathway complex. However, in *M. maripaludis*, both archaella and pili are located peritrichously on the cell surface, leaving open the question of how these archaea are able to differentiate their type IV pilin-like proteins in terms of signal peptide removal and N-glycosylation and what role this difference has in assembly of the two surface organelles.

## 4. Conclusions

*M. maripaludis* possesses two surface appendages—type IV-like pili and archaella—that are believed to be assembled by a bacterial type IV pili mechanism. Both archaellins and pilins are type IV pilin-like proteins with class III signal peptides which are cleaved by distinct prepilin peptidase-like enzymes. In addition, both kinds of subunits are modified with similar but unique N-linked glycans that depend on the action of the oligosaccharyltransferase AglB. In this report, we demonstrate that the two posttranslational modifications of archaellins can occur independently of each other, whereas for pilins, the N-glycosylation does not occur unless the signal peptide has been cleaved first. How the cells are able to distinguish the two types of type IV pilin-like molecules in this way is unknown.

## Supplementary Materials

Supplementary materials can be accessed at: http://www.mdpi.com/2075-1729/5/1/85/s1.
