# Supplementary material for: Pilin Processing Follows a Different Temporal Route than That of Archaellins in Methanococcus maripaludis
Source: Life (Basel). 2015 Jan 5;5(1):85–101. doi: 10.3390/life5010085 (PMC4390842; doi:10.3390/life5010085)
Supplement: Supplementary file 1 [file life-05-00085-s001.pdf]

Supplementary Material

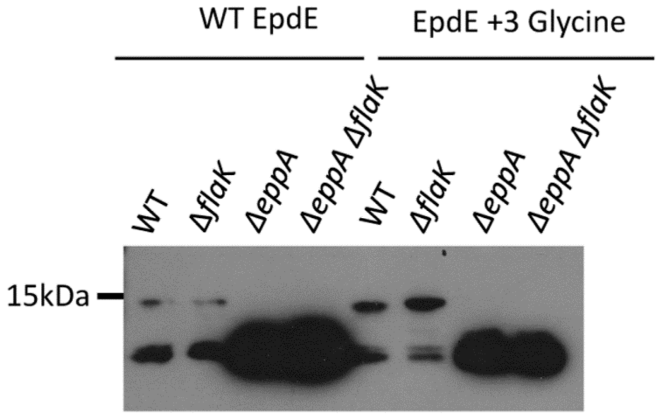

Figure S1. Western blot analysis of various *M. maripaludis* mutant strains expressing a plasmid borne C-terminal FLAG-tagged version of wildtype EpdE or EpdE with the +3 amino acid position (relative to the signal peptide cleavage site) changed to glycine. Whole cell lysates were separated by SDS-PAGE (17.5% gel), transferred to Immobilon membrane and developed with antibodies to the FLAG-tag. No bands were detected in Western blots of cells lacking the plasmid carrying the FLAG-tagged pilin (not shown).

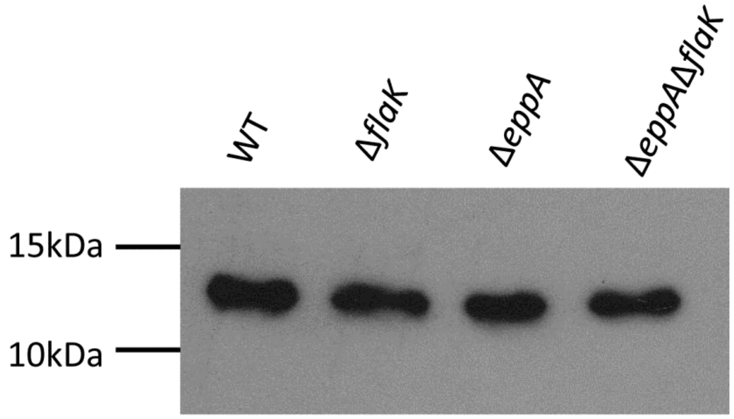

Figure S2. Western blot analysis of various *M. maripaludis* mutant strains expressing a plasmid borne C-terminal FLAG-tagged version of EpdE with the +1 amino acid (relative to the signal peptide cleavage site) changed to alanine and the +3 amino acid position changed to glycine. Whole cell lysates were separated by SDS-PAGE (17.5% gel), transferred to Immobilon membrane and developed with antibodies to the FLAG-tag.

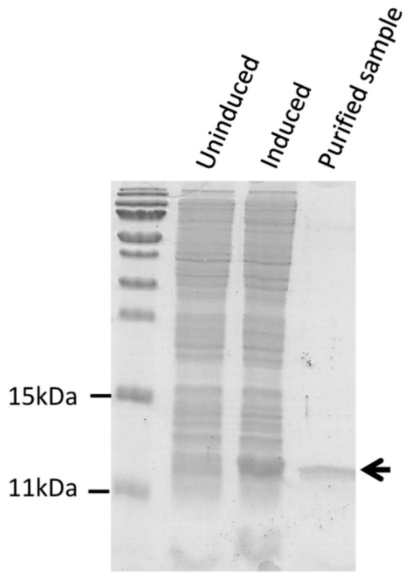

Figure S3. Purification of C-terminal Histagged EpdE by Ni-affinity chromatography. Log phase *E. coli* strain BL21 (DE3)/pLysS cells containing pKJ900 were induced by the addition of IPTG for 2 hours. Histagged EpdE was purified from induced cells using nickel affinity purification under denaturing conditions. Purified EpdE, indicated by the arrow, was further concentrated for use as antigen for antibody generation in chickens.

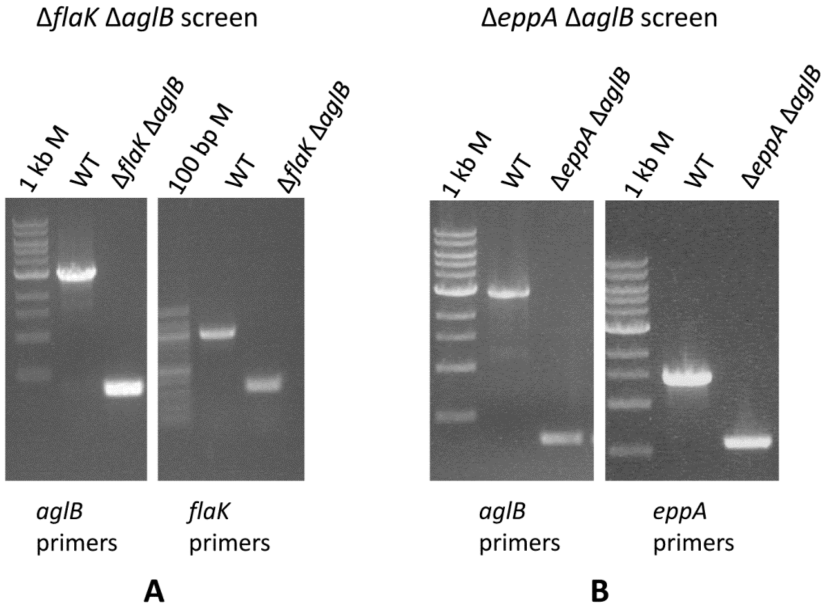

Figure S4. Confirmation of the creation of the double gene deletion strains by PCR. (**A**) PCR screening of *aglB* and *flaK* in the double mutant Δ*flaK*Δ*aglB* using whole cells of the wildtype and the deletion strain as template with gene specific primers. In each case, the first lane shows the PCR product obtained when wildtype cells were used as template and the second lane shows the result using the deletion strain as the template. (**B**) PCR screening of *aglB* and *eppA* in the double mutant Δ*eppA*Δ*aglB* using whole cells of wildtype and the deletion strain as template. In each case, the first lane shows the PCR product obtained when the wildtype cells were used as template and the second lane shows the PCR product obtained when the deletion strain was used as template.
